# Outcomes of beta blocker use in advanced hepatocellular carcinoma treated with immune checkpoint inhibitors

**DOI:** 10.3389/fonc.2023.1128569

**Published:** 2023-02-14

**Authors:** Y. Linda Wu, Grace van Hyfte, Umut Özbek, Marlene Reincke, Anuhya Gampa, Yehia I. Mohamed, Naoshi Nishida, Brooke Wietharn, Suneetha Amara, Pei-Chang Lee, Bernhard Scheiner, Lorenz Balcar, Matthias Pinter, Arndt Vogel, Arndt Weinmann, Anwaar Saeed, Anjana Pillai, Lorenza Rimassa, Abdul Rafeh Naqash, Mahvish Muzaffar, Yi-Hsiang Huang, Ahmed O. Kaseb, Masatoshi Kudo, David J. Pinato, Celina Ang

**Affiliations:** ^1^ Division of Hematology and Medical Oncology, Tisch Cancer Institute, Icahn School of Medicine at Mount Sinai, New York, NY, United States; ^2^ Department of Population Health Science and Policy, Icahn School of Medicine at Mount Sinai, New York, NY, United States; ^3^ Department of Medicine II, Faculty of Medicine, Medical Center University of Freiburg, Freiburg, Germany; ^4^ Section of Gastroenterology, Hepatology and Nutrition, The University of Chicago Medical Center, Chicago, IL, United States; ^5^ Department of Gastrointestinal Medical Oncology, The University of Texas MD Anderson Cancer Center, Houston, TX, United States; ^6^ Department of Gastroenterology and Hepatology, Kindai University Faculty of Medicine, Osaka, Japan; ^7^ Division of Medical Oncology, Department of Medicine, Kansas University Cancer Center, Kansas City, KS, United States; ^8^ Division of Hematology/Oncology, East Carolina University, Greenville, NC, United States; ^9^ Division of Gastroenterology and Hepatology, Taipei Veterans General Hospital, National Yang-Ming University, Taipei, Taiwan; ^10^ Division of Gastroenterology and Hepatology, Department of Internal Medicine III, Medical University of Vienna, Vienna, Austria; ^11^ Department of Gastroenterology, Hepatology and Endocrinology, Hannover Medical School, Hannover, Germany; ^12^ Department of Internal Medicine, University Medical Center of the Johannes Gutenberg University Mainz, Mainz, Germany; ^13^ Department of Biomedical Sciences, Humanitas University, Pieve Emanuele (Milan), Italy; ^14^ Medical Oncology and Hematology Unit, Humanitas Cancer Center, IRCCS Humanitas Clinical and Research Hospital, Rozzano (Milan), Italy; ^15^ Division of Cancer Treatment and Diagnosis, National Cancer Institute, Bethesda, MD, United States; ^16^ Department of Surgery and Cancer, Imperial College London, Hammersmith Hospital London, London, United Kingdom

**Keywords:** hepatocellular carcinoma, immune checkpoint inhibitors, cancer immunotherapy, beta-adrenergic blockade, beta blocker

## Abstract

**Background:**

In patients with cirrhosis, portal hypertension increases intestinal permeability, dysbiosis, and bacterial translocation, promoting an inflammatory state that can lead to the progression of liver disease and development of hepatocellular carcinoma (HCC). We aimed to investigate whether beta blockers (BBs), which can mediate portal hypertension, conferred survival benefits in patients treated with immune checkpoint inhibitors (ICIs).

**Methods:**

We conducted a retrospective, observational study of 578 patients with unresectable HCC treated with ICI from 2017 to 2019 at 13 institutions across three continents. BB use was defined as exposure to BBs at any time during ICI therapy. The primary objective was to assess the association of BB exposure with overall survival (OS). Secondary objectives were to evaluate the association of BB use with progression-free survival (PFS) and objective response rate (ORR) according to RECIST 1.1 criteria.

**Results:**

In our study cohort, 203 (35%) patients used BBs at any point during ICI therapy. Of these, 51% were taking a nonselective BB. BB use was not significantly correlated with OS (hazard ratio [HR] 1.12, 95% CI 0.9-1.39, *P* = 0.298), PFS (HR 1.02, 95% CI 0.83-1.26, *P* = 0.844) or ORR (odds ratio [OR] 0.84, 95% CI 0.54-1.31, *P* = 0.451) in univariate or multivariate analyses. BB use was also not associated with incidence of adverse events (OR 1.38, 95% CI 0.96-1.97, *P* = 0.079). Specifically, nonselective BB use was not correlated with OS (HR 0.94, 95% CI 0.66-1.33, *P* = 0.721), PFS (HR 0.92, 0.66-1.29, *P* = 0.629), ORR (OR 1.20, 95% CI 0.58-2.49, *P* = 0.623), or rate of adverse events (OR 0.82, 95% CI 0.46-1.47, *P* = 0.510).

**Conclusion:**

In this real-world population of patients with unresectable HCC treated with immunotherapy, BB use was not associated with OS, PFS or ORR.

## 1 Introduction

Hepatocellular carcinoma (HCC) is a leading cause of cancer death worldwide and often diagnosed in advanced stages when cure is no longer feasible (1). For patients with advanced HCC, multikinase inhibitors such as sorafenib and lenvatinib had long been the first-line systemic therapy but offered poor outcomes and high toxicity (2). Recently, the combination of atezolizumab, a programmed death-ligand 1 (PD-L1) inhibitor, and bevacizumab was shown to improve overall survival (OS) compared to sorafenib in patients with unresectable HCC in the IMbrave150 trial (3, 4). In addition, the phase III HIMALAYA trial recently showed that the combination of durvalumab and tremelimumab had superior efficacy to sorafenib in the first-line treatment of unresectable HCC (5). Even in patients who had received multikinase inhibitors in the front line, treatment with immunotherapy on progression of disease may induce a response (6). As a result, immune checkpoint inhibitors (ICIs) have now supplanted multikinase inhibitors as standard of care front line therapy for advanced HCC. However, advanced HCC still carries a poor prognosis, and response to ICIs is limited, underscoring the need to identify markers of ICI response.

Increasingly, there is interest in understanding drug-drug interactions in the context of cancer immunotherapy. In particular, common concomitant medications such as antibiotics, steroids, antacids, metformin, and opioids that may have immunomodulatory effects have been investigated in order to examine their potential role in either enhancing ICI efficacy or contributing to toxicity (7). The disruption of the gut microbiome, through antibiotic use, for example, has been associated with decreased ICI efficacy and impaired T cell antitumor response (8, 9). In HCC, a recent study found that patients who responded to ICI had greater gut microbial diversity than non-responders, providing further evidence that the gut microbiome may impact response to ICI (10).

The interaction of the gut microbiome and ICI therapy has important implications for patients with HCC. Liver cirrhosis is well-known to underlie HCC carcinogenesis, and portal hypertension (pHTN) promotes progression of liver disease through immune activation: pHTN causes splanchnic vasodilation and pathological angiogenesis, increasing intestinal permeability and dysbiosis, which leads to bacterial translocation and induces a pro-inflammatory state (11). Both pHTN and chronic inflammation are risk factors for the development of HCC and tumor progression (12). Therefore, it is possible that the attenuation of pHTN may decrease aberrant neoangiogenesis and bacterial translocation-mediated inflammation driving HCC tumorigenesis and progression.

Beta blockers (BBs), particularly non-selective BBs, are standard prophylaxis for patients with cirrhosis and pHTN-induced varices (13). They have been shown to modulate pHTN-associated dysbiosis through a reduction in intestinal bacterial overgrowth, intestinal permeability, and bacterial translocation (14, 15). Additionally, in preclinical studies, BBs decrease tumor cell proliferation, proinflammatory cytokine load, and catecholamine-driven angiogenesis (16–18). Some clinical studies suggest that BB use is associated with lower incidence of HCC in patients with cirrhosis (19–21). One nationwide population-based study in Taiwan found that propranolol use improved OS in patients with unresectable or metastatic HCC who were treated with sorafenib, locoregional therapy, or radiotherapy (22). However, there is a paucity of data addressing the effect of beta blockade on outcomes of patients with advanced HCC in the era of immunotherapy. We aimed to evaluate whether BB use conferred survival benefits in patients treated with ICIs using real-world data.

## 2 Methods

### 2.1 Study population

The study population consisted of 578 patients with unresectable HCC treated with ICI from 2017 to 2019 at 13 institutions across North America (*N* = 247), Europe (*N* = 240), and Asia (*N* = 91). Patients included in this study had a diagnosis of HCC in accordance with American Association for the Study of Liver Disease (23) and European Association for the Study of the Liver (24) guidelines, received systemic ICI therapy (either monotherapy or in combination), and had measurable disease according to RECIST 1.1 criteria at the start of ICI. All patients were treated according to routine clinical practice, including prescriptions for BB. The decision to start ICI therapy was made at the discretion of the treating physician based on current evidence-based practice guidelines, institutional standards, and often after multidisciplinary tumor board discussions.

### 2.2 Study design

Patient demographics and clinical data, including Barcelona Clinic Liver Cancer (BCLC) stage, Child-Pugh (CP) class, Eastern Cooperative Oncology Group (ECOG) performance status, alpha fetoprotein (AFP) level, presence of cirrhosis (clinically or radiologically diagnosed), etiology of liver disease, type and duration of ICI therapy, type and indication of BB use, duration of BB use, follow-up and vital status, were collected retrospectively. Baseline data were defined at the time of ICI initiation, and treatment response was evaluated through radiologic staging of the disease using computerized tomography and/or magnetic resonance imaging approximately every 9 weeks during treatment. BB use was defined as exposure at any time during ICI therapy. BBs were classified as nonselective (propranolol, nadolol, carvedilol, labetalol) and cardio-selective (metoprolol, atenolol, bisoprolol, nebivolol), and standard doses were used. Indications for BB use were evaluated and included variceal prophylaxis, cardiovascular disease, and other indications.

The primary outcome was to evaluate the association between BB use and OS, measured from the time of ICI initiation until date of death from any cause or date of last follow-up. Secondary outcomes included assessing the effect of BB use on objective response rate (ORR), defined as the proportion of patients with either radiographic complete response (CR) or partial response (PR), duration of response (DOR), defined as best response of CR, PR, or stable disease (SD), progression-free survival (PFS), measured from the time of ICI initiation until radiographic progression, and development of treatment-related adverse events (AEs) of any grade. All responses were evaluated according to RECIST 1.1 criteria. AEs were defined based on the Common Terminology Criteria for Adverse Events (CTCAE) classification, version 5.0, and identified based on investigator review of clinical notes, radiographic, and laboratory data. Evaluation of BB exposure was based on the presence of an active prescription in the medical record per clinical notes or medication records. Baseline BB use was defined as exposure within 30 days prior to ICI initiation, and concurrent BB use was defined as exposure between the dates of ICI initiation and cessation.

### 2.3 Statistical analysis

Patient characteristics were summarized descriptively with medians and interquartile ranges for continuous variables and frequencies and proportions for categorical variables. Categorical variables were examined across BB exposure levels utilizing either chi-square tests or Fisher’s exact tests, where appropriate, while the nonparametric Mann-Whitney U test was used for continuous variables. Univariable and multivariable Cox proportional hazard models were fitted for OS and PFS. Univariable and multivariable logistic regression models were generated to evaluate the association of the aforementioned variables with ORR, the presence of any AE, and for AEs graded 2 or higher. Covariates were selected for the multivariable models if they were found to be significant in univariable analysis. Each model is summarized with hazard ratios (HR) or odds ratios (OR) and their coinciding 95% confidence intervals (CIs). Additional subgroup analyses were performed in order to examine the interaction between each covariate and BB exposure. A forest plot was generated which summarizes the subgroup analyses with interaction term and associated p-value. For all survival analyses the proportional hazards assumption was tested and found to be satisfied. Variance inflation factors in multivariable models were below 5 to indicate an absence of multicollinearity. The level of significance was maintained at 0.05. All analyses were carried out in R version 4.1.2 (Vienna, Austria).

## 3 Results

### 3.1 Baseline characteristics

Baseline clinical characteristics are reported in **Table 1**. The majority of the cohort were male (*N* = 464, 80%), with a median age of 65 years (IQR: 58-70 years). Most patients (*N* = 406, 70%) had radiologic or pathologic evidence of cirrhosis at baseline. The causes of underlying liver disease were hepatitis C virus (*N =* 209, 36%), hepatitis B virus (*N* = 125, 22%), alcohol-related (*N* = 120, 21%), and nonalcoholic steatohepatitis (NASH)-associated (*N* = 75, 13%). Most patients had preserved liver function with CP class A disease (*N* = 413, 74%) and good performance status with ECOG score either 0 (*N* = 300, 52%) or 1 (*N* = 259, 45%).

**Table 1:** Baseline characteristics by beta blocker exposure.

		No BB Exposure (%)	BB Exposure (%)	*P* value	All Patients (%)
***N***		375	203		578
**Age (years), median**		64	66		65
**Male**		302 (80.5)	162 (79.8)	0.9194	464 (80.3)
**Region**	**USA**	150 (40.0)	97 (47.8)	<0.0001	247 (42.7)
	**Europe**	145 (38.7)	95 (46.8)		240 (41.5)
	**Asia**	80 (21.3)	11 (5.4)		91 (15.7)
**Cirrhosis**		248 (66.1)	158 (77.8)	0.0045	406 (70.2)
**Etiology**	**HBV**	98 (26.1)	27 (13.3)	0.0005	125 (21.6)
	**HCV**	132 (35.2)	77 (37.9)	0.5744	209 (36.2)
	**EtOH**	71 (18.9)	49 (24.1)	0.1722	120 (20.8)
	**NASH**	45 (12.0)	30 (14.8)	0.4196	75 (13.0)
	**Other**	16 (4.3)	20 (9.9)	0.0141	36 (6.2)
**BCLC Stage**	**A**	7 (1.9)	7 (3.4)	0.4378	14 (2.4)
	**B**	52 (13.9)	25 (12.3)		77 (13.3)
	**C**	314 (83.7)	168 (82.8)		482 (83.4)
**Child Pugh Class**	**A**	286 (78.1)	127 (64.8)	0.0016	413 (72.3)
	**B**	78 (21.3)	67 (34.2)		145 (25.4)
	**C**	2 (0.5)	2 (1.0)		4 (0.7)
**ECOG PS**	**0**	210 (56.0)	90 (44.3)		300 (51.9)
	**1**	161 (42.9)	98 (48.3)		259 (44.8)
	**2**	2 (0.5)	15 (7.4)		17 (2.9)
	**3**	2 (0.5)	0.0		2 (0.3)
**Portal Vein Thrombosis**		109 (29.9)	70 (37.2)	0.0063	179 (32.4)
**Extrahepatic Metastasis**		203 (54.1)	106 (52.2)	0.7236	309 (53.5)
**Baseline AFP > 400**		152 (41.5)	75 (37.3)	0.3595	227 (40.0)
**Immunotherapy**	**PD-1 alone**	281 (75.1)	154 (75.9)	0.4777	435 (75.3)
	**PD-1/** **CTLA-4**	28 (7.5)	9 (4.4)		37 (6.4)
	**PD-1/TKI**	27 (7.2)	15 (7.4)		42 (7.3)
	**Other**	38 (10.2)	25 (12.3)		63 (10.9)
**First-Line ICI**		174 (46.4)	90 (44.3)	0.6978	264 (45.7)
**Prior Systemic Therapy**		200 (53.3)	112 (55.2)	0.7368	312 (54.0)
**Prior Local Therapy**		250 (66.7)	130 (64.0)	0.5868	380 (65.7)
**Previous Liver Resection**		133 (35.5)	53 (26.1)	0.0274	186 (32.2)
**Antibiotic Exposure**		126 (34.8)	94 (49.2)	0.0013	220 (39.7)
**Antacid Exposure**		145 (50.5)	88 (55.3)	0.3801	233 (52.2)

BB, beta blocker; HBV, hepatitis B virus; HCV, hepatitis C virus; EtOH, alcohol use; NASH, nonalcoholic steatohepatitis; BCLC, Barcelona Clinic Liver Cancer; ECOG, Eastern Cooperative Oncology Group; PS, performance status; AFP, alpha fetoprotein; TKI, tyrosine kinase inhibitor; ICI, immune checkpoint inhibitor.

At the time of initiation of ICI, 482 patients (83%) had BCLC stage C disease. The majority of patients (*N* = 435, 75%) treated with ICIs received a PD-1 inhibitor alone. ICI was given in the first-line in 46% of patients (*N* = 264), and 54% of patients (*N* = 312) received at least one prior systemic therapy. Many patients (*N* = 380, 66%) received prior locoregional therapy, with transarterial chemoembolization being the most common (*N* = 258, 45%). In addition, 186 patients (32%) had undergone prior surgical resection.

### 3.2 Treatment outcomes

During a median follow-up of 30.8 months (IQR: 17.2-40.3 months), there were 360 deaths (62%) noted. A total of 541 patients could be evaluated for best radiographic response to ICI therapy per RECIST 1.1 criteria. There were 36 patients with CR (6.7%), 78 with PR (14.4%), and 216 with SD (39.9%), which correspond with an ORR of 21.1% and disease control rate (DCR) of 61.0%. At the time of analysis, the median duration of ICI therapy was 4.1 months (IQR: 1.9-9.3 months). Progression of disease was the most common cause of ICI discontinuation (*N* = 303, 52%).

Treatment-related AEs developed in 336 patients (58%), but only 97 patients (17%) developed grade 3 or higher events. The most common AEs were fatigue (*N* = 127, 22%), skin toxicity (*N* = 100, 17%), and liver toxicity (*N* = 96, 17%), with 142 (25%) experiencing other AEs, such as cytopenias, nausea, fever or infections, neuropathy, and electrolyte imbalances (**Table 2**). The most common grade 3 or higher AE was hepatotoxicity (*N* = 33, 6%), followed by fatigue (*N* = 13, 2.2%) and colitis (*N* = 13, 2.2%), with 37 patients (6%) experiencing other grade 3 or higher AEs (**Table 2**). A total of 37 patients (6.4%) also experienced bleeding events, 16 (43%) of which were gastrointestinal or variceal bleeding.

**Table 2:** Adverse events.

Adverse Event	Any Grade, N (%)	Grade 3 or Above, N (%)
Skin	100 (17.3%)	11 (1.9%)
Diarrhea/Colitis	52 (9.0%)	13 (2.2%)
Fatigue	127 (22.0%)	13 (2.2%)
Hepatitis/Liver Toxicity	96 (16.6%)	33 (5.7%)
Thyroid Toxicity	34 (5.9%)	1 (0.2%)
Pituitary Toxicity	10 (1.7%)	3 (0.5%)
Rheumatologic Toxicity	11 (1.9%)	4 (0.7%)
Lung Toxicity	37 (6.4%)	6 (1.0%)
Hypertension	37 (6.4%)	4 (0.7%)
Proteinuria	20 (3.5%)	0 (0%)
Other	142 (24.6%)	37 (6.4%)

### 3.3 Beta blocker exposure

Two hundred and three (35%) patients had BB use at any point during ICI therapy, of which only 4 (2%) patients had used BB up to the time of ICI initiation but not concurrently with ICI. Conversely, 22 (11%) patients started BB after ICI was initiated. However, most patients (*N* = 177, 87%) had been on BB before the start of ICI and continued on immunotherapy. Furthermore, BBs were long-term medications for patients who had been on BBs prior to ICI, with 96% (*N* = 173) being prescribed for more than 4 weeks.

The types of BBs used were evenly divided: 51% (*N* = 103) of patients were on a nonselective BB and 49% (*N* = 100) were taking a cardio-selective BB. Of those taking a nonselective BB, the indication was predominantly for variceal prophylaxis (*N* = 69, 67%), followed by cardiovascular indications (*N* = 32, 31%), with 2 unclear indications. As expected, cardio-selective BBs were prescribed for cardiovascular indications (*N* = 96, 96%), except for 2 patients who had a cardio-selective BB for variceal prophylaxis and 2 more for other indications.

Overall, the baseline characteristics between patients with or without BB exposure were comparable, but there were some exceptions (**Table 1**). Patients who had BB exposure were more often from the United States or Europe, had a history of cirrhosis, neoplastic portal vein thrombosis (PVT), and antibiotic exposure. Patients exposed to BBs and antibiotics were most commonly treated with beta-lactams, quinolones, or cephalosporins, typically for a single week-long course for fever of unknown origin and early in the course of ICI therapy (within 30 days). The effect of antibiotic therapy on ICI outcomes in this cohort of HCC patients was previously evaluated (25). Patients without BB exposure tended to have HBV as an etiology of liver disease, CP class A disease, and a history of prior resection to treat HCC.

### 3.4 Association of baseline beta blocker use with immunotherapy outcomes

**Figure 1:**
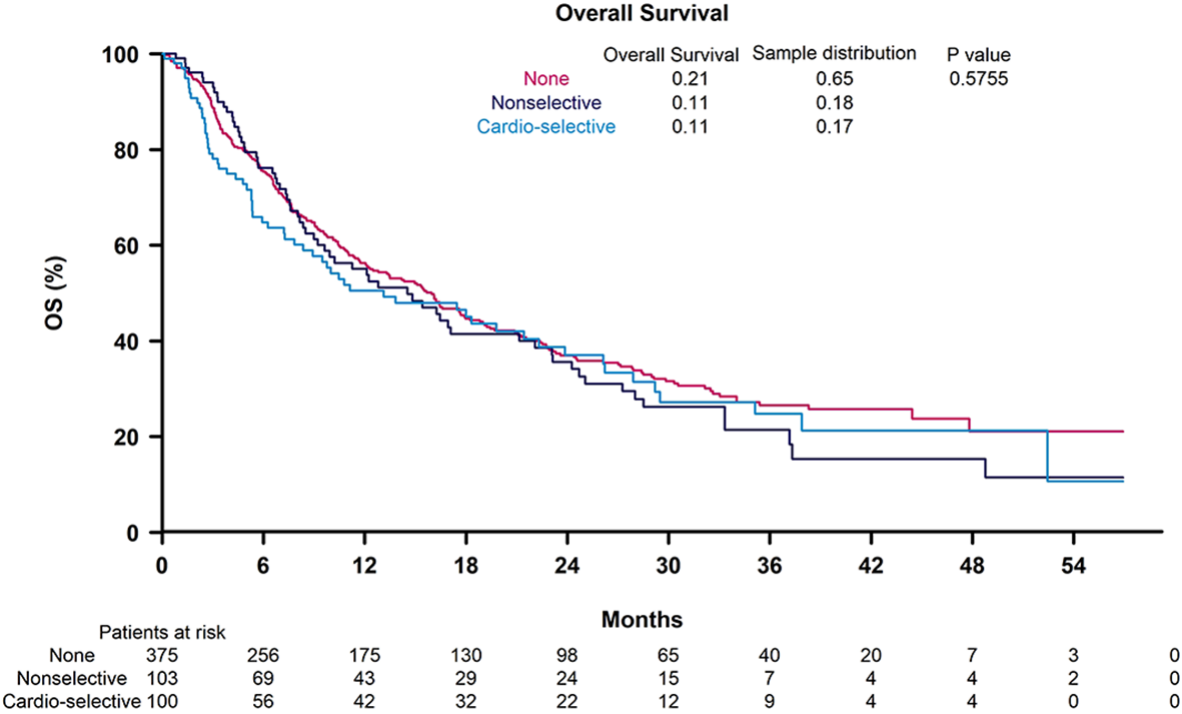
Kaplan-Meier curves for overall survival according to non-selective beta blocker (BB) use, cardioselective BB use, and no BB use.

**Figure 2:**
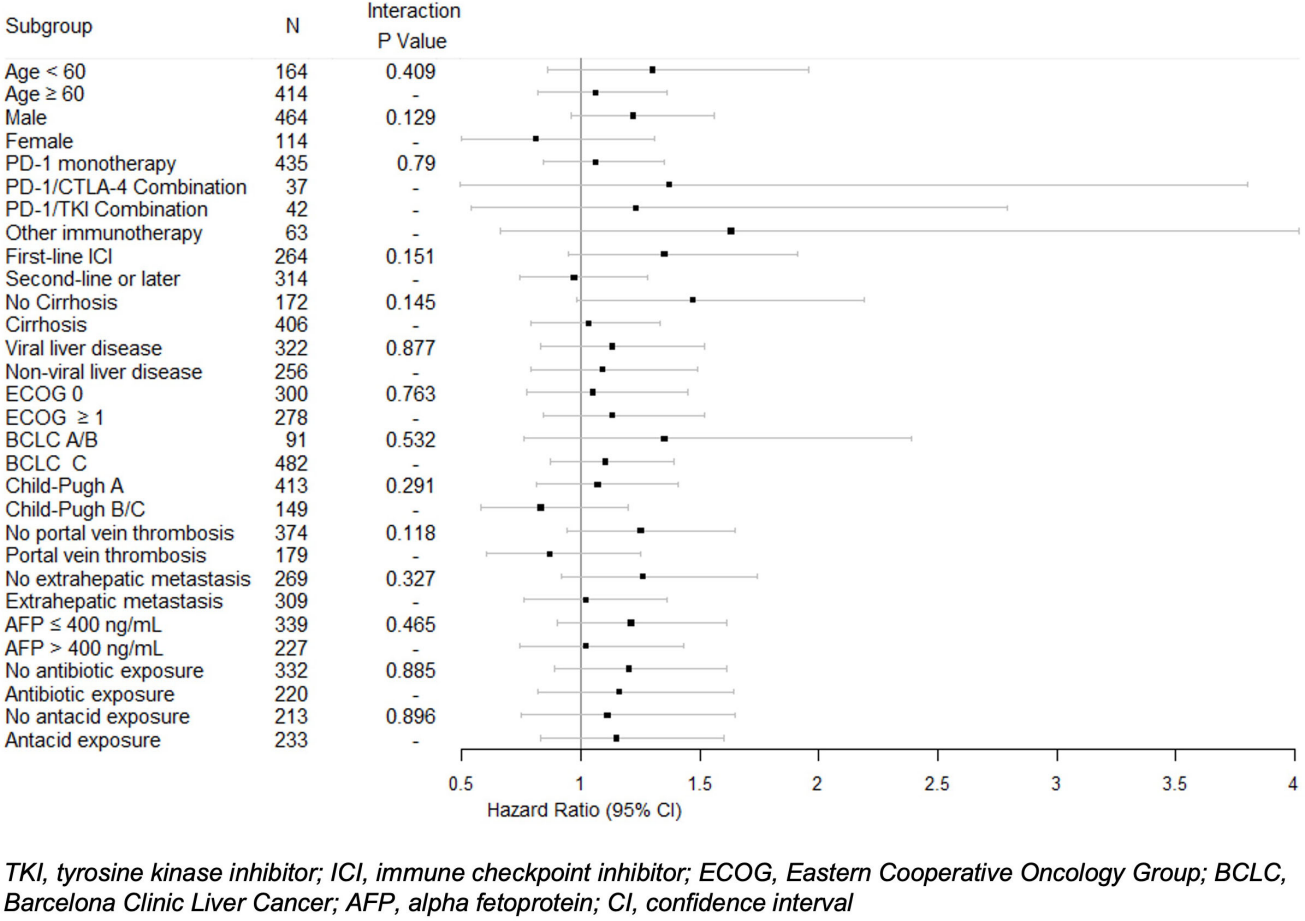
Univariable Cox proportional hazard model of overall survival with interactions between beta blocker exposure and subgroups. Hazard ratios and 95% confidence intervals for beta blocker exposure are shown with *P* values for each subgroup.

In univariable analysis, BB use was not significantly correlated with OS (HR 1.12, 95% CI 0.9-1.39) (**Table 3**). Nonselective BB type was also not associated with OS (HR 0.94, 95% CI 0.66-1.33), and these results are illustrated in **Figure 1**. Variables that were associated with improved OS included CP class A (HR 0.51, 95% CI 0.41-0.64) and performance status ECOG 0 (HR 0.69, 95% CI 0.56-0.84). Factors contributing to worsened OS included presence of neoplastic PVT (HR 1.93, 95% CI 1.55-2.41) and AFP > 400 (HR 1.59, 95% CI 1.29-1.96). Other baseline characteristics tested that did not have associations with OS included age, sex, cirrhosis, viral etiology of liver disease, and presence of extrahepatic metastases. Multivariable analyses identified the same independent predictors of OS, including CP class A disease (HR 0.55, 95% CI 0.44-0.70), presence of neoplastic PVT (HR 1.60, 95% CI 1.27-2.02), and AFP > 400 (HR 1.39, 95% CI 1.11-1.74), but not ECOG 0 (HR 0.81, 95% CI 0.65-1). The effect of BB exposure on OS was not affected by multiple variables evaluated in subgroup analyses (**Figure 2**), including age, sex, ICI monotherapy vs. combination therapy, line of therapy, cirrhosis, performance status, stage, liver function, presence of neoplastic PVT, extrahepatic metastasis, or AFP level. In particular, given the possible immunomodulatory effects of BBs, concomitant exposure to antibiotics and antacids was examined but not found to influence the effect of BB use on OS.

**Table 3:** Univariable and multivariable Cox proportional hazard models for overall survival.

Predictor	Univariable HR (95% CI)	*P* Value	Multivariable HR (95% CI)	*P* Value
Age	1.00 (0.99, 1.01)	0.473		
Sex	1.02 (0.79, 1.32)	0.887		
Cirrhosis	0.93 (0.75, 1.17)	0.559		
Viral etiology	0.86 (0.7, 1.06)	0.167		
Child-Pugh A	0.51 (0.41, 0.64)	<0.001	0.55 (0.44, 0.70)	<0.001
ECOG 0	0.69 (0.56, 0.84)	<0.001	0.81 (0.65, 1)	0.053
Portal vein thrombosis	1.93 (1.55, 2.41)	<0.001	1.60 (1.27, 2.02)	<0.001
Extrahepatic metastasis	1.04 (0.84, 1.28)	0.735		
AFP > 400	1.59 (1.29, 1.96)	<0.001	1.39 (1.11, 1.74)	0.004
BB exposure	1.12 (0.9, 1.39)	0.298		
Nonselective BB	0.94 (0.66, 1.33)	0.721		

HR, hazard ratio; CI, confidence interval; ECOG, Eastern Cooperative Oncology Group; AFP, alpha fetoprotein; BB, beta blocker.

Next, the PFS was evaluated and results are tabulated in **Table 4**. Univariable analysis did not find a significant correlation between BB exposure and PFS (HR 1.02, 95% CI 0.83-1.26). Again, nonselective BB use was not determined to be associated with PFS (HR 0.92, 95% CI 0.66-1.29). Variables associated with PFS included CP class A disease (HR 0.71, 95% CI 0.57-0.89), presence of neoplastic PVT (HR 1.41, 95% CI 1.14-1.76), and performance status ECOG 0 (HR 0.77, 95% CI 0.63-0.94). On multivariable analyses, independent predictors of PFS were CP class A disease (HR 0.74, 95% CI 0.58-0.94) and presence of neoplastic PVT (HR 1.37, 95% CI 1.09-1.72). The cirrhosis status and CP class did not significantly affect the PFS of patients with BB exposure in subgroup analyses.

**Table 4:** Univariable and multivariable Cox proportional hazard models for progression-free survival.

Predictor	Univariable HR (95% CI)	*P* Value	Multivariable HR (95% CI)	*P* Value
Age	0.99 (0.98, 1)	0.109		
Sex	1.04 (0.81, 1.32)	0.774		
Cirrhosis	0.91 (0.73, 1.13)	0.380		
Viral etiology	0.94 (0.77, 1.15)	0.530		
Child-Pugh A	0.71 (0.57, 0.89)	0.003	0.74 (0.58, 0.94)	0.015
ECOG 0	0.77 (0.63, 0.94)	0.012		
Portal vein thrombosis	1.41 (1.14, 1.76)	0.002	1.37 (1.09, 1.72)	0.008
Extrahepatic metastasis	1.08 (0.89, 1.32)	0.441		
AFP > 400	1.21 (0.99, 1.48)	0.065		
BB exposure	1.02 (0.83, 1.26)	0.844		
Nonselective BB	0.92 (0.66, 1.29)	0.629		

HR, hazard ratio; CI, confidence interval; ECOG, Eastern Cooperative Oncology Group; AFP, alpha fetoprotein; BB, beta blocker.

We then evaluated whether BB exposure was associated with ICI response (**Table 5**). Univariable analyses showed that BB use was also not significantly correlated with ORR (OR 0.84, 95% CI 0.54-1.31). Nonselective BB use did not play a role in objective response (OR 1.20, 95% 0.58-2.49). The other characteristics evaluated, including age, sex, presence of cirrhosis, viral etiology of liver disease, CP class, presence of neoplastic PVT, performance status, presence of extrahepatic metastases, and AFP level were not found to be associated with response to ICI. Subgroup analyses of the effect of BBs on ORR revealed no significant effect of cirrhosis or CP class.

**Table 5:** Univariable hazard model for objective response.

Predictor	Univariable OR (95% CI)	*P* Value
Age	1.00 (0.98, 1.02)	0.967
Sex	0.72 (0.41, 1.26)	0.251
Cirrhosis	0.95 (0.61, 1.48)	0.816
Viral etiology	1.03 (0.68, 1.57)	0.877
Child-Pugh A	0.85 (0.53, 1.37)	0.512
ECOG 0	1.20 (0.79, 1.82)	0.386
Portal vein thrombosis	1.03 (0.65, 1.62)	0.914
Extrahepatic metastasis	1.03 (0.68, 1.56)	0.894
AFP > 400	1.19 (0.78, 1.81)	0.424
BB exposure	0.84 (0.54, 1.31)	0.451
Nonselective BB	1.20 (0.58, 2.49)	0.623

OR, odds ratio; CI, confidence interval; ECOG, Eastern Cooperative Oncology Group; AFP, alpha fetoprotein; BB, beta blocker.

Finally, the effect of BBs on development of AEs was evaluated. BB exposure was not associated with development of any AE (OR 1.38, 95% CI 0.96-1.97). No statistically significant benefit of BB exposure against bleeding events was observed (OR 1.83, 95% CI 0.94-3.58). Only 4 patients in the cohort developed ascites while treated with ICI: 2 patients who had prior BB exposure and 2 who did not. The presence of neoplastic PVT was found to increase the risk of any AE in univariable analysis (OR 1.53, 95% CI 1.05-2.22) and was an independent predictor of AE development in multivariable analysis (OR 1.56, 95% CI 1.06, 2.31) (**Table 6**). Conversely, patients with a viral etiology of HCC were less likely to develop any AE according to univariable analysis (OR 0.56, 95% CI 0.40-0.79) and multivariable analysis (OR 0.52, 95% CI 0.36-0.75).

**Table 6:** Univariable and multivariable Cox proportional hazard models for any adverse events.

Predictor	Univariable OR (95% CI)	*P* Value	Multivariable OR (95% CI)	*P* Value
Age	1.00 (0.98, 1.01)	0.834		
Sex	1.27 (0.84, 1.96)	0.271		
Cirrhosis	0.82 (0.57, 1.19)	0.296		
Viral etiology	0.56 (0.40, 0.79)	0.001	0.52 (0.36, 0.75)	<0.001
Child-Pugh B or C	0.70 (0.48, 1.03)	0.070		
ECOG 1-3	1.07 (0.76, 1.49)	0.701		
Portal vein thrombosis	1.53 (1.05, 2.22)	0.025	1.56 (1.06, 2.31)	0.025
Extrahepatic metastasis	1.37 (0.98, 1.91)	0.069		
AFP > 400	1.08 (0.77, 1.53)	0.654		
BB exposure	1.38 (0.96, 1.97)	0.079		
Nonselective BB	0.82 (0.46, 1.47)	0.510		

OR, odds ratio; CI, confidence interval; ECOG, Eastern Cooperative Oncology Group; AFP, alpha fetoprotein; BB, beta blocker.

## 4 Discussion

In this multicenter, international observational study, we evaluated the effect of BB use in patients with advanced HCC treated with ICI and found no significant association between BB exposure and OS. No significant associations were observed between BB exposure and secondary outcomes, including PFS, ORR, and development of AEs.

The effect of BB exposure on outcomes in HCC had previously been investigated, with some evidence that BB use may improve survival in patients with HCC. One Swedish study of 2104 patients in a national cancer registry between 2006 and 2015 found that BB use, particularly nonselective BB use, was associated with a lower mortality rate, but the mortality benefit appeared to be limited to patients with localized disease (26). Similarly, a small retrospective study of 36 patients with non-metastatic HCC who had undergone surgical resection or locoregional therapy found that BB use failed to predict HCC recurrence but was associated with improved OS after these curative interventions (27). As described earlier, a Taiwanese nationwide study of 4680 patients with unresectable and metastatic HCC from 2000 to 2013 found that propranolol reduced the risk of mortality from HCC, but no significant difference in recurrence-free survival (RFS) was observed in the 867 patients with localized disease (22). However, it is also worthwhile to note that these studies were conducted before ICIs gained widespread use as front line therapy for advanced HCC.

To the best of our knowledge, our study is the first to evaluate the effect of BB use in HCC treated with immunotherapy. Since the IMbrave150 trial changed the treatment paradigm for patients with advanced HCC, it has become more important to develop prognostic markers and to understand the impact of common concomitant medications on response to treatment. The effect of BB use is of particular clinical relevance as many patients with HCC take BBs at baseline as standard of care for variceal prophylaxis and sometimes for cardiovascular indications (28). Biologically, the role of β-adrenergic signaling has been well described pre-clinically to modulate the tumor microenvironment (TME) and response to immunotherapy (29), and there is significant interest in validating these findings in the clinical setting.

Cancer cells have been shown to express β-adrenergic receptors (βARs) (30), and adrenergic signaling has been linked to tumorigenesis and cancer progression through promotion of processes such as DNA repair, oncogene activation, inflammation and immune response, angiogenesis, survival, and epithelial-mesenchymal transition (29). Mouse models have been used to mechanistically show the effect of stress on the TME in various solid tumors. For example, Bucsek et al. showed that chronic stress in mice induced by cold exposure increased intratumoral noradrenaline, which subsequently reduced intratumoral CD8^+^ T cell frequency and functionality (31). Conversely, the addition of propranolol reduced βAR signaling, which converted tumors to an immunologically active TME with increased CD8^+^ T cell frequency and effector phenotype, decreased expression of PD-1, and elevated effector CD8^+^ T cell to CD4^+^ regulatory T cell ratio, leading to increased efficacy of anti-PD-1 checkpoint blockade (31). Kokolus et al. also showed that βAR blockade enhanced the antitumor effect of anti-PD-1 checkpoint inhibitor in a murine model of melanoma (32). Recently, in mouse models of sarcoma and colon cancer, propranolol reduced tumor angiogenesis, increased T cell infiltration, and reduced myeloid-derived suppressor cell infiltration, leading to an up-regulation of PD-L1 on tumor-associated macrophages, ultimately enhancing the efficacy of anti-CTLA4 therapy (33). These preclinical studies suggest that beta blockade may improve response to ICIs.

In the clinical setting, while no study before ours has evaluated the association between BB exposure and ICIs in HCC, it has been explored in other cancers, and thus far, results have been inconclusive but largely negative. In melanoma, BBs were found to have no independent prognostic effect on RFS in a recent phase III trial of adjuvant pembrolizumab in patients with high-risk stage III resected melanoma (34). Similarly, in a retrospective study of advanced melanoma, concurrent BB use in patients treated with ICI did not affect ORR, PFS, or OS (35). In contrast, one retrospective analysis of 195 patients with metastatic melanoma treated with ICI found that nonselective BB exposure improved survival compared to no BB use and β_1_-selective antagonist use (32). Another retrospective study of 109 patients with non-small cell lung cancer (NSCLC) treated with ICIs demonstrated that BB use may be associated with improved PFS but not OS (36). Another study in NSCLC analyzed the effect of multiple concomitant medications with ICIs and found that baseline BB use was not associated with clinical outcomes (37). A recent meta-analysis of 13 aggregated studies in mostly melanoma, NSCLC, and renal cell carcinoma showed that concurrent BB use with immunotherapy was not significantly associated with improved survival (38). Our negative findings add to growing evidence that the effect of BBs on clinical outcomes after treatment with ICIs may be limited despite preclinical data, suggesting that multiple interdependent pathways likely modulate the TME in humans and that there is a need to account for differences in experimental and real-world observations.

The unique pathophysiology of liver cirrhosis and microbial dysbiosis also add complexity to understanding of the TME and HCC tumorigenesis, suggesting that beta blockade is unlikely to mediate these interactions in easily predictable ways. BBs have both hemodynamic and non-hemodynamic mechanisms of action. Previously, nonselective BBs have been shown to increase intestinal transit, reducing bacterial overgrowth and translocation, in patients with cirrhosis independently of their hemodynamic functions (14, 39). While dysbiosis is known to contribute to HCC tumorigenesis, the strategy for targeting the gut microbiota-liver axis is still unclear. It is also unclear how beta blockade changes the microbiome in humans. Additionally, the ways in which ICIs can affect the microbiome are not well-characterized, though the composition of gut microbiota has been shown in preclinical studies to influence immunotherapy efficacy through regulation of immune responses (40). Our study also assessed whether antacids and antibiotics modified the effect of BB use but found no survival differences. These results are consistent with prior data in HCC demonstrating that antibiotic and antacid exposure during ICI therapy did not affect OS (25, 41) but are in opposition with studies conducted in other solid tumors (42), which may indicate that the HCC microbiome has immunomodulatory effects distinct from that of other cancers. While nonselective BBs may reduce bacterial overgrowth and translocation, further studies are need to better understand whether BB use changes the HCC microbiome and how this may affect outcomes with ICI therapy.

Our study also showed that BB exposure in patients with advanced HCC treated with ICIs did not increase the development of any AEs. On the other hand, and more surprisingly, use of BB also did not reduce the number of bleeding events observed. Given the real-world population evaluated in this cohort, a limitation of our study included the inconsistent reporting of the presence or absence of esophageal varices, as not all patients underwent pretreatment esophagogastroduodenoscopy (EGD). Although the decision to perform an EGD was not standardized, it was made on a case-by-case basis by the treating physician, in line with routine clinical practice. As such, the baseline degree of pHTN could not be fully characterized in the full patient cohort. Regardless, concomitant BB use did not affect the rate of bleeding events or development of ascites, which is in turn consistent with its lack of correlation with clinical outcomes after ICI therapy. In the subgroup analysis of patients with neoplastic PVT, BB use did not confer a statistically significant survival benefit. Given the low rate of bleeding events in the cohort (6.4%), it is possible that the study was underpowered to detect any influence of BB exposure. In the overall analysis, only the presence of neoplastic PVT was identified as an independent predictor of AE development, whereas a viral etiology of HCC was linked with a reduced risk of AE. The presence of neoplastic PVT is a well-known negative prognosticator of HCC and a criterion for classification into advanced stage disease (43), and the increased risk of AE is likely reflective of worse liver function. Further, our findings that viral etiologies of HCC did not increase incidence of AE is supported by prior studies confirming the safety and tolerability of ICIs in patients with HBV and HCV in HCC and other solid tumors (44, 45). In fact, patients with viral etiologies had a lower incidence of AEs, and while the cause is not entirely clear, it is possible that patients with viral HCC are diagnosed and initiate treatment when liver disease is less advanced as these patients are more likely to receive screening and treatment for liver disease prior to diagnosis of HCC. Studies are underway to better understand the outcomes of patients with viral HCC.

This is the first study investigating the effect of BB exposure in patients with advanced HCC treated with immunotherapy using real-world data. Although other studies of BB use in patients receiving immunotherapy for solid tumors have produced inconsistent results, our findings add to the body of evidence that BB use is not associated with improved survival outcomes. Overall, our international, multicenter cohort offers broad generalizability and is reflective of a diverse, real-world population, including patients with more advanced liver disease (CP classes B and C) who are typically excluded from clinical trial participation. Collection of detailed patient characteristics also allowed us to control for multiple possible confounding factors, such as the patient’s age, performance status, liver function, disease stage, and HCC risk factor.

However, the study also had several limitations, including those inherent to retrospective cohort studies that require validation in prospective studies. Patients taking BBs at baseline likely have increased comorbid conditions, including cirrhosis and cardiovascular disease, that may increase their risk of mortality. We accounted for these possible confounders by controlling for baseline liver function and performance status. However, the OS evaluated in this study was only reflective of all-cause mortality, and the specific cause of death was often unavailable or inconsistently documented in the medical record. While the effect of other potentially confounding concomitant medications such as antibiotics and antacids were assessed, other medications such as aspirin, statins, metformin, and steroids that may affect survival outcomes in HCC were not examined in this study (46–49). In addition, due to the observational design, the definition of BB exposure did not include dose and duration, and BB adherence could not be confirmed based on review of medical records alone. The number of patients in our study may also have been too small to detect an association between BB use and OS, particularly when subdivided by BB type and duration. Finally, our cohort does not have a large proportion of patients treated with the IMbrave150 regimen consisting of atezolizumab and bevacizumab that has now become standard of care for advanced HCC, and prior studies in colon cancer suggest a favorable effect of BB use in bevacizumab-containing therapy (50). However, studies are currently underway to assess the prognostic impact of concomitant medications on treatment outcomes with this combination.

In conclusion, in our retrospective cohort of patients with unresectable HCC treated with ICI, no statistically significant differences in OS, PFS, or ORR were observed between patients who used BBs and those who did not. Concomitant BB use was safe and did not increase the risk of AEs. Further prospective and larger observational studies, as well as mechanistic studies, are needed to elucidate the effect of beta blockade on HCC and its interaction with the microbiome and immune activation.

## Data availability statement

The raw data supporting the conclusions of this article will be made available by the authors, without undue reservation.

## Ethics statement

The studies involving human participants were reviewed and approved by the Institutional Review Board (IRB) at Imperial College London, whose review was accepted by all participating institutions’ IRBs. Written informed consent for participation was not required for this study in accordance with the national legislation and the institutional requirements.

## Author contributions

YLW and CA designed the study. YLW conducted the investigation, analysis, interpretation of data, and wrote the original draft. GvH and UÖ contributed to data analysis and methodology. MR, AG, YM, NN, BW, SA, P-CL, BS, LB, MP, AV, AW, AS, AP, LR, AN, MM, YHH, AK, MK, and DP contributed to the investigation and data curation. DP and CA supervised the study. All authors contributed to the article and approved the submitted version.
